# Increased transferase ratio is associated with adverse cardio-cerebral events in patients with unstable angina: A retrospective cohort study

**DOI:** 10.1097/MD.0000000000034563

**Published:** 2023-08-04

**Authors:** Dong Lv, Yanfu Guo, Xia Li, Li Zhang

**Affiliations:** a Department of Cardiology, Beijing Renhe Hospital, Beijing, China; b Graduate school of Jiamusi University, Heilongjiang, China; c Department of Cardiology, Hegang People’s Hospital, Heilongjiang, China; d Jiamusi University, Heilongjiang, China; e The Central Hospital of Jiamusi City, Heilongjiang, China.

**Keywords:** alanine aminotransferase, aspartate aminotransferase, outcome, percutaneous coronary intervention, unstable angina

## Abstract

To investigate the prognostic role of the elevated aspartate and alanine aminotransferase (AST/ALT) ratio in patients with unstable angina (UA). In this observational study, all patients with UA undergoing percutaneous coronary intervention at our center from January 2019 to December 2020 were examined. Clinical presentations, laboratory parameters, and procedural characteristics were collected. The primary endpoint was a composite of major adverse cardio-cerebral events (MACCE), such as death, nonfatal myocardial infarction, nonfatal stroke, and target vessel revascularization. In total, 1123 eligible UA patients were enrolled in the present study (mean age 62.3 years; 54.5% of male). Patients in the upper tertile of the AST/ALT ratio were older, had more extensive coronary stenosis, and had poor nutritional status (*P* < .05). Meanwhile, the cumulative incidence of MACCE at 13 months of follow-up increased in a stepwise manner and across the tertile of the AST/ALT ratio, predominantly driven by target vessel revascularization (both log-rank *P* < .001). Importantly, the AST/ALT ratio was associated with MACCE in a multivariate analysis that was adjusted for potential covariates (hazard ratio 1.72, 95% confidence interval 1.48–1.99, *P* < .01). The optimal cutoff point of the AST/ALT ratio to predict MACCE was 1.29 (area under the curve 0.77, 95% confidence interval 0.69–0.84, *P* < .001), with sensitivity and specificity of 77.5% and 65.1%, respectively. The increased AST/ALT ratio, especially when above 1.29, is associated with MACCE in patients with UA undergoing percutaneous coronary intervention.

## 1. Introduction

Aminotransferase, such as the alanine aminotransferase (ALT) and aspartate aminotransferase (AST), is widely used for the routine diagnosis marker for liver injury. It is known that ALT is highly expressed in the liver, while AST is abundantly expressed in the kidney, muscle, and brain.^[1]^ Fernando De Ritis, for the first time, has proposed the AST/ALT ratio (also named the De Ritis ratio) as a useful indicator for viral hepatitis.^[2]^ Subsequently, this serological index was used to assess alcoholic and nonalcoholic liver disease, autoimmune liver diseases, hepatitis C, and hepatic fibrosis.^[3–6]^

The heart–liver interaction in patients with atrial fibrillation, heart failure, and myocardial infarction (MI) was observed in previous studies.^[7–9]^ Recently, the markedly increased AST/ALT ratio, which was commonly used to reflect the liver injury, was found to be associated with several cardiovascular diseases (CVD), e.g., total coronary occlusion,^[10]^ and even mortality in the patients presenting with ST-segment elevation myocardial infarction.^[11]^ Furthermore, the predictive value of the AST/ALT ratio on mortality was also confirmed in the stable coronary artery disease (CAD) patients after the treatment of percutaneous coronary intervention (PCI).^[12]^ Although the outcome predictive value of the AST/ALT ratio has been reported in patients with MI and stable CAD, its prognosis in unstable angina (UA) is still unknown. Therefore, in this study using Chinese UA patients, we aimed to investigate the relationship between the AST/ALT ratio and the major adverse cardio-cerebral events (MACCE).

## 2. Material and methods

### 2.1. Study population

We retrospectively screened consecutive patients who were admitted for unstable angina undergoing PCI from January 2019 to December 2020 at Beijing Renhe Hospital, Beijing, China. Patients were excluded from this study if they had: (1) age < 18 or >80 years old; (2) left ventricular ejection fraction <30%; (3) severe liver dysfunction (i.e., AST or ALT > 120 U/L, total bilirubin > 51 µmol/L or albumin < 28 g/L); (4) severe kidney dysfunction (i.e., creatinine > 186 µmol/L) or (5) incomplete data. The protocol of this study was in accordance with the Declaration of Helsinki and approved by the Ethics Committee of Beijing Renhe Hospital.

### 2.2. Coronary angiography and intervention

Clinical management and invasive coronary procedure were performed according to current guidelines.^[13]^ Briefly, diagnostic coronary angiography was performed using a 5F or 6F catheter (1 F = 0.33 mm) using the transradial or transfemoral approach. In the visual evaluation of the baseline coronary angiogram, obstructive CAD was defined as an epicardial coronary stenosis ≥50%, and nonobstructive CAD was defined as luminal stenosis 1 to 49%.^[14]^ To assess the extent and severity of CAD, the Gensini score was calculated based on baseline angiographic findings for each study participant.^[15]^ The decision of PCI was made at the discretion of the interventional cardiologist.

### 2.3. Data collection

Through chart review, the trained physicians collected the following information of patients: clinical presentations, medical histories, echocardiographic findings, laboratory results, procedural characteristics, and medications at discharge. Body mass index (kg/m^2^) was calculated as weight divided by height squared. The blood samples were obtained from all enrolled patients in the morning after fasting for 12 hours overnight. Biochemical indexes of these blood samples (e.g., transaminase), were measured in the core laboratory of our hospital prior to the coronary angiography procedure.

### 2.4. Clinical outcomes

The primary outcome of this study was MACCE, consisting of all-cause death, nonfatal myocardial infarction (MI), nonfatal stroke, and target vessel revascularization (TVR). Secondary outcomes were all-cause death, nonfatal MI, nonfatal stroke, TVR, and a composite outcome of all-cause death, nonfatal MI, and nonfatal stroke (i.e., “hard endpoint”). MI was defined as an increase in cardiac troponin I levels above the 99th percentile upper-reference limit accompanied by new ischemic symptoms, electrocardiographic or echocardiographic changes.^[16]^ Of note, peri-procedural (i.e., type 4–5) MI was not considered as an adverse outcome. The stroke was determined by a neurological specialist according to the presence of neurologic deficits and related imaging findings. TVR referred to performing myocardial revascularization for the stenotic coronary vessel at the baseline of angiogram. The population enrolled in this study was followed up through an office interview, telephone, or reviewing medical records.

### 2.5. Statistical analysis

The study population was divided into tertiles according to their serum AST/ALT ratio. Continuous variables were shown as mean ± standard deviation or median (interquartile range). Continuous variables were compared between tertiles using the one-way ANOVA or Kruskal–Wallis H test. Categorical variables were expressed as numbers (percentages) and compared using the Chi-square test or Fisher exact test. The cumulative incidence of clinical outcomes was assessed using Kaplan–Meier estimates and compared between tertiles using the log-rank test. The relationship between the AST/ALT ratio (shown as continuous or categorical variables) and clinical outcomes was analyzed by univariate analysis or multivariate models using Cox proportional hazards analysis. The results were presented as a hazard ratio and the corresponding 95% confidence interval. The variables included in the multivariate models were either with a *P -v*alue < 0.1 in univariate analysis or clinically relevant to clinical outcomes. The receiver operating characteristic (ROC) curve was performed to explore the diagnostic value and optimal threshold of the AST/ALT ratio for MACCE. The Harrell C-index was calculated to evaluate the incremental effect of AST, ALT, and AST/ALT ratio on prognosis predicting value. Meanwhile, the Youden index was used to determine sensitivity and specificity of the diagnosis of MACCE. A two-sided *P-v*alue < .05 was considered statistically significant and all statistical analyzes were performed using SPSS 20.0 software (IBM, Armonk, NY) and the R Programming Language (version 3.6.3).

## 3. Results

### 3.1. Patient characteristics

Chart review was initially performed in 1173 patients and 50 patients were excluded from the current study (i.e., 32 patients were >80 years old, 6 patients had reduced left ventricular ejection fraction, 12 patients had severe liver or kidney dysfunction). Finally, a total of 1123 eligible UA patients undergoing PCI were enrolled in this study. The mean age of the enrolled patients was 62.3 years and 54.5% of them were men (Table 1). Hypertension (76.5%), type 2 diabetes mellitus (40.6%), and current smoking (44.4%) were common cardiovascular risk factors in this population. The median AST/ALT ratio was 1.13, and tertile cutoff points of the AST/ALT ratio were 0.96 and 1.31, respectively (Table 2). In particularly, 87.1% of the patients had obstructive CAD on coronary angiogram, and almost two-thirds patients had left anterior descending artery disease, and one-fourth patients had 3-vessel disease. Finally, nearly half of the patients underwent PCI with a drug-eluting stent (Table 3).

**Table 1:** Baseline characteristics of the study population.

	Overall N = 1123	AST/ALT ratio			*P*-value
		Lowest tertile	Middle tertile	Highest tertile	
		n = 368	n = 371	n = 384	
Demographics					
Age, years	62.3 ± 9.5	58.9 ± 9.6	62.5 ± 9.0	65.3 ± 8.9	<.01
Male, N (%)	612 (54.5)	221 (60.1)	190 (51.2)	201 (52.3)	.03
BMI, kg/m^2^	25.6 ± 2.7	25.9 ± 2.7	25.6 ± 2.8	25.4 ± 2.7	.05
Current smoking, N (%)	499 (44.4)	174 (47.3)	149 (40.2)	176 (45.8)	.12
Clinical presentation					
Systolic BP, mm Hg	146.1 ± 22.0	144.9 ± 20.0	147.7 ± 22.5	145.8 ± 23.3	.21
Diastolic BP, mm Hg	82.4 ± 12.9	84.2 ± 12.5	82.6 ± 12.9	80.6 ± 13.1	<.01
Heart rate, bpm	78.4 ± 15.2	78.9 ± 13.8	77.9 ± 15.8	78.4 ± 16.0	.70
Medical histories, N (%)					
Hypertension	859 (76.5)	271 (73.6)	296 (79.8)	292 (76.0)	.14
Type 2 diabetes mellitus	456 (40.6)	151 (41.0)	164 (44.2)	141 (36.7)	.11
Prior stroke	311 (27.7)	101 (27.4)	100 (27.0)	110 (28.6)	.87
CAD	377 (33.6)	113 (30.7)	121 (32.6)	143 (37.2)	.150
Prior myocardial infarction	55 (4.9)	18 (4.9)	15 (4.0)	22 (5.7)	.57
Prior revascularization	173 (15.4)	54 (14.7)	60 (16.2)	59 (15.4)	.85
Echocardiography					
LVEF, %	65.2 ± 8.0	65.7 ± 7.3	64.9 ± 8.3	65.2 ± 8.4	.39
LVEDD, mm	46.3 ± 4.1	46.6 ± 3.9	46.1 ± 4.1	46.3 ± 4.3	.35
IVSD, mm	9.0 ± 1.5	9.1 ± 1.5	9.0 ± 1.4	9.0 ± 1.7	.306

ALT = alanine transaminase, AST = aspartate aminotransferase, BMI = body mass index, BP = blood pressure, CAD = coronary artery disease, IVSD = interventricular septal thickness at diastole, LVEDD = left ventricular end diastolic diameter, LVEF = left ventricular ejection fraction.

**Table 2:** Laboratory parameters of the study population.

	Overall N = 1123	AST/ALT ratio			*P*-value
		Lowest tertile	Middle tertile	Highest tertile	
		n = 368	n = 371	n = 384	
AST/ALT§	0.18–9.49	0.18–0.96	0.96–1.31	1.31–9.49	–
Albumin, g/L	40.6 ± 3.9	41.0 ± 3.7	40.7 ± 4.0	40.2 ± 3.9	.01
Total bilirubin, umol/L	13.4 ± 5.5	13.3 ± 5.6	13.5 ± 5.6	13.5 ± 5.5	.84
Creatinine, umol/L	64.3 ± 17.2	63.9 ± 16.8	63.7 ± 16.3	65.2 ± 18.4	.41
Uric acid, umol/L	313.0 (249.0, 371.0)	319.5 (257.3, 377.5)	307.0 (242.0, 371.0)	305.0 (236.3, 365.8)	.03
Fasting glucose, mmol/L	6.0 (5.3, 7.5)	6.2 (5.5, 7.9)	6.0 (5.4, 7.5)	5.7 (5.1, 6.9)	<.01
Total triglyceride, mmol/L	1.6 (1.1, 2.4)	1.7 (1.2, 2.6)	1.6 (1.1, 2.4)	1.4 (1.0, 2.1)	.03
Total cholesterol, mmol/L	4.8 ± 1.3	4.9 ± 1.2	4.9 ± 1.3	4.7 ± 1.3	.27
LDL-C, mmol/L	3.0 ± 0.9	3.0 ± 0.8	3.0 ± 0.9	2.9 ± 0.9	.43
C-reactive protein, mg/L	1.5 (0.7, 3.8)	1.7 (0.9, 4.3)	1.5 (0.7, 3.8)	1.3 (0.6, 3.5)	.08
White blood cell, *10^9^/L	6.5 ± 1.5	6.5 ± 1.5	6.4 ± 1.5	6.4 ± 1.4	.42
Hemoglobin, g/L	134.4 ± 16.7	136.1 ± 17.8	134.8 ± 15.4	132.5 ± 16.7	.01
Platelet, *10^9^/L	208.5 ± 57.6	207.5 ± 55.6	211.4 ± 59.4	206.5 ± 57.6	.46

ALT = alanine transaminase, AST = aspartate transaminase, LDL-C = low density lipoprotein cholesterol.

§ Data shown as minimal-maximal value.

**Table 3:** Angiographic and procedural characteristics of the study population.

	Overall N = 1123	AST/ALT ratio			*P*-value
		Lowest tertile	Middle tertile	Highest tertile	
		n = 368	n = 371	n = 384	
Coronary angiography					
Severity of CAD, N (%)					<.01
No disease	11 (1.0)	3 (0.8)	3 (0.8)	5 (1.3)	
Nonobstructive disease	134 (11.9)	62 (16.8)	42 (11.3)	30 (7.8)	
Obstructive disease	978 (87.1)	303 (82.3)	326 (87.9)	349 (90.9)	
Extent of obstructive CAD, N (%)					
Left main disease	39 (3.5)	10 (2.7)	16 (4.3)	13 (3.4)	.49
Three-vessel disease	269 (24.0)	72 (19.6)	94 (25.3)	103 (26.8)	.05
Gensini score	23.0 (14.0, 35.0)	20.0 (13.0, 32.0)	24.0 (15.0, 38.0)	24.0 (14.0, 36.0)	.02
Location of obstructive CAD, N (%)					
Left main	39 (3.5)	10 (2.7)	16 (4.3)	13 (3.4)	.49
Left anterior descending	775 (69.0)	241 (65.5)	262 (70.6)	272 (70.8)	.21
Left circumflex	494 (44.0)	137 (37.2)	173 (46.6)	184 (47.9)	<.01
Right coronary artery	539 (48.0)	151 (41.0)	181 (48.8)	207 (53.9)	<.01
Coronary intervention					
Any intervention, N (%)	626 (55.7)	192 (52.2)	211 (56.9)	223 (58.1)	.23
Stent implantation, N (%)	569 (50.7)	179 (48.6)	195 (52.6)	195 (50.8)	.57
Number of stent per patient	0.8 ± 1.0	0.8 ± 1.0	0.8 ± 1.0	0.8 ± 1.0	.57

ALT = alanine transaminase, AST = aspartate aminotransferase, CAD = coronary artery disease.

Compared to the patients with lowest tertile of the AST/ALT ratio, patients with a higher AST/ALT ratio were older, more likely to have MI, obstructive CAD, and 3-vessel CAD (all *P* < .05) (Table 3). Meanwhile, in contrast to the patients with lower tertile of the AST/ALT ratio, patients with the higher AST/ALT ratio had lower serum albumin, uric acid, glucose, total triglycerides, and hemoglobin concentrations (all *P* < .05) (Table 2). Other clinical characteristics, laboratory parameters, procedural details, and medications were comparable among the tertiles of the AST/ALT ratio (Tables 1–4).

**Table 4:** Medications at discharge of the study population.

N (%)	Overall N = 1123	AST/ALT ratio			*P*-value
		Lowest tertile	Middle tertile	Highest tertile	
		n = 368	n = 371	n = 384	
Aspirin	1085 (96.6)	355 (96.5)	358 (96.5)	372 (96.9)	.96
Clopidogrel	574 (51.1)	185 (50.3)	192 (51.8)	197 (51.3)	.92
Ticagrelor	143 (12.7)	46 (12.5)	50 (13.5)	47 (12.2)	.88
Nitrates	921 (82.0)	307 (83.4)	310 (83.6)	304 (79.2)	.21
β-Blocker	580 (51.6)	179 (48.6)	186 (50.1)	215 (56.0)	.10
Calcium antagonist	558 (49.7)	172 (46.7)	197 (53.1)	189 (49.2)	.22
Statins	1109 (98.8)	362 (98.6)	368 (99.2)	379 (98.7)	.78
Ezetimibe	215 (19.1)	68 (18.5)	66 (17.8)	81 (21.1)	.48
ACEI or ARB	544 (48.4)	177 (48.1)	186 (50.1)	181 (47.1)	.71
Sacubitril/valsartan	14 (1.2)	6 (1.6)	5 (1.3)	3 (0.8)	.53
SGLT-2 inhibitor	33 (2.9)	10 (2.7)	12 (3.2)	11 (2.9)	.93
DPP4 inhibitor	265 (23.6)	89 (24.2)	95 (25.7)	81 (21.1)	.33
Diuretic	38 (3.4)	13 (3.5)	11 (3.0)	14 (3.6)	.86

ACEI = angiotensin-converting enzyme inhibitor, ALT = alanine transaminase, ARB = angiotensin receptor blocker, AST = aspartate aminotransferase, DPP4 = dipeptidyl peptidase 4 inhibitor, SGLT-2 = sodium-dependent glucose transporters 2.

### 3.2. Clinical outcomes

All enrolled patients had clinical follow-up, with a median follow-up duration of 13 months. MACCE, the primary outcome of this study, occurred in 40 (3.6%) patients, and was mainly driven by TVR (2.0%). One patient died in the patients with top tertile of the AST/ALT ratio due to cancer. Compared to the patients with lowest tertile of the AST/ALT ratio, patients with an increased AST/ALT ratio also had an increased risk of stroke, TVR, the composite outcome of death, MI, stroke, as well as MACCE (*P* < .05) (Table 5).

**Table 5:** Clinical outcomes of the study population.

	Overall N = 1123	AST/ALT ratio			*P*-value
		Lowest tertile	Middle tertile	Highest tertile	
		n = 368	n = 371	n = 384	
Length of follow-up, months	13.0 (12.0, 14.0)	12.9 (11.9, 14.1)	12.8 (12.0, 13.9)	13.0 (12.1, 14.1)	.380
Clinical outcomes, N (%)					
All-cause death	1 (0.1)	0	0	1 (0.3)	1.000
Nonfatal MI	4 (0.4)	0	2 (0.5)	2 (0.5)	.555
Nonfatal stroke	14 (1.2)	1 (0.3)	4 (1.1)	9 (2.3)	.032
TVR	23 (2.0)	1 (0.3)	2 (0.5)	20 (5.2)	<.001
Death, MI or stroke	19 (1.7)	1 (0.3)	6 (1.6)	12 (3.1)	.006
MACCE	40 (3.6)	2 (0.5)	8 (2.2)	30 (7.8)	<.001

ALT = alanine transaminase, AST = aspartate aminotransferase, MACCE = major adverse cardiac and cerebrovascular event, MI = myocardial infarction, TVR = target vessel revascularization.

**Figure 1.**
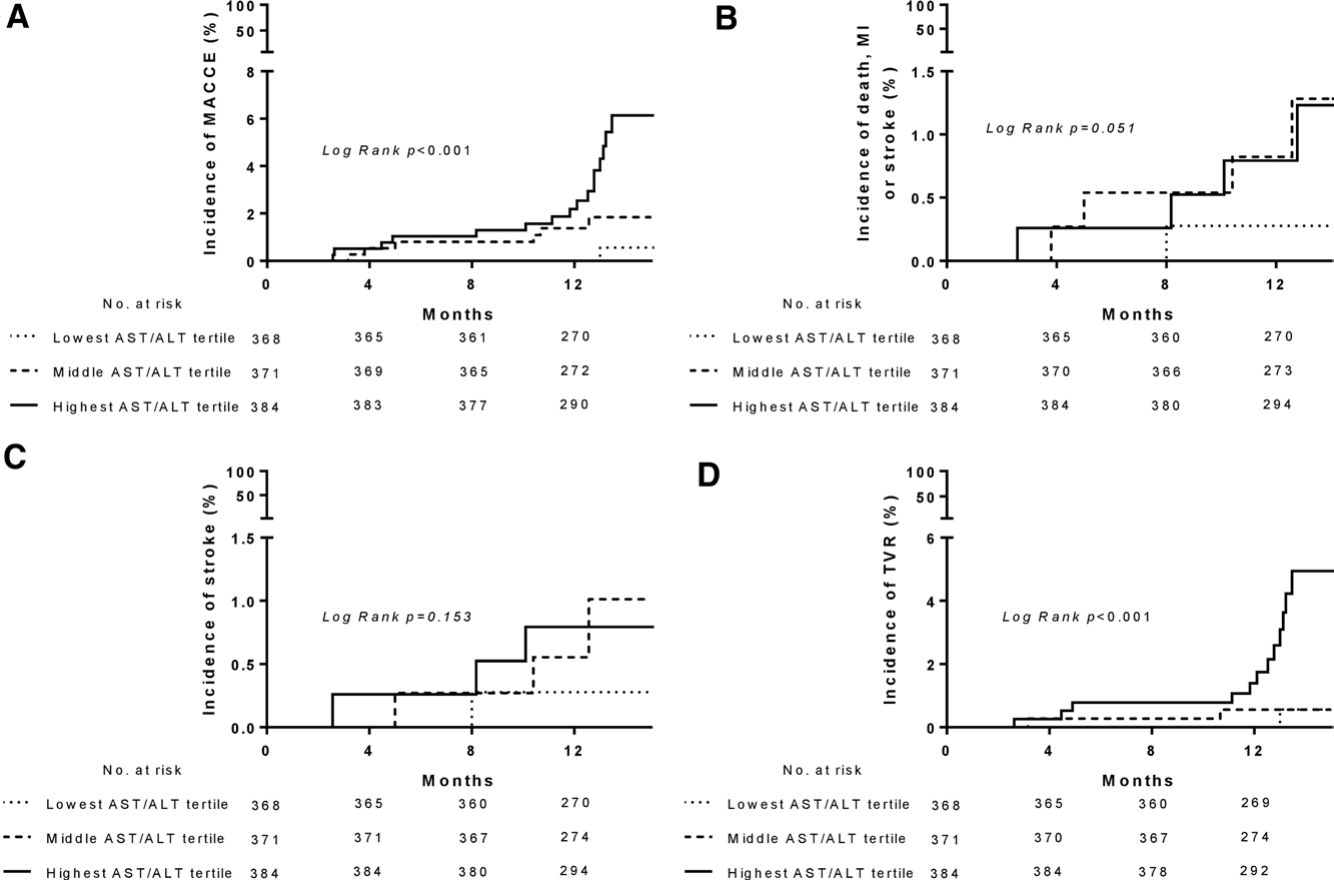
Cumulative incidence of major adverse cardio-cerebral events (MACCE) (A), death, myocardial infarction (MI) or stroke (B), stroke (C), and target vessel revascularization (TVR) (D) across tertiles of the aspartate and alanine aminotransferase (AST/ALT) ratio.

As shown in Figure 1, the cumulative incidence of MACCE and TVR at 13 months of follow-up markedly increased in a stepwise manner in the tertiles of the AST/ALT ratio (both log-rank *P* < .001). However, this stepwise elevation pattern is not found with respect to the stroke or the composite outcome of death, and MI (both log-rank *P* > .05) (Fig. 1).

### 3.3. Multivariate analysis

In univariate analysis, high AST/ALT ratio (modulated as continuous variable or category variable), high levels of C-reactive protein, high Gensini score, and low hemoglobin levels were significantly associated with the elevated risk of MACCE (all *P* < .05). Furthermore, using Cox proportional-hazards analysis adjusting for potential confounders (e.g., age, Gensini score, and hemoglobin), high AST/ALT ratio (either modulated as a continuous variable or category variable), high C-reactive protein levels were consistently associated with increased incidence of MACCE (all *P* < .05) (Table 6). Meanwhile, we found that the addition of the AST/ALT ratio, in comparison with AST or ALT, showed the maximum enhancement on prognosis predicting value for MACCE on the basis of the baseline model including age, male, hemoglobin, C-reactive protein, and Gensini score (baseline model +AST/ALT ratio vs +AST vs. +ALT, Harrell C-index 0.78 vs 0.71 vs 0.72).

**Table 6:** Predictors of MACCE in the Cox proportional hazards analysis.

	Univariate analysis		Multivariate analysis		Multivariate analysis	
	HR (95%CI)	*P*-value	HR (95%CI)	*P*-value	HR (95%CI)	*P*-value
Age	1.02 (0.98–1.06)	.38	1.00 (0.96–1.04)	.93	0.99 (0.95–1.03)	.68
Male	0.97 (0.52–1.84)	.93	1.11 (0.56–2.21)	.77	1.19 (0.61–2.33)	.62
Hemoglobin	0.97 (0.94–0.99)	.007	0.98 (0.96–1.01)	.13	0.99 (0.96–1.01)	.22
C-reactive protein	1.03 (1.01–1.04)	<.01	1.02 (1.01–1.03)	<.01	1.02 (1.01–1.03)	<.01
Gensini score	1.01 (1.00–1.03)	.01	1.01 (1.00–1.02)	.15	1.01 (1.00–1.02)	.08
AST/ALT ratio						
Continuous variable	1.80 (1.57–2.06)	<.01	1.72 (1.48–1.99)	<.01	–	–
Category variable						
Lowest tertile	–	–	–	–	–	–
Middle tertile	4.99 (1.05–23.70)	.04	–	–	4.60 (0.96–21.90)	.05
Highest tertile	11.58 (2.76–48.60)	<.01	–	–	10.01 (2.30–43.55)	<.01

HR of continuous variable referring to an increase per one standard deviation.

ALT = alanine transaminase, AST = aspartate aminotransferase, CI = confidence interval, HR = hazard ratio.

### 3.4. Optimal prognostic threshold of the AST/ALT ratio

**Figure 2.**
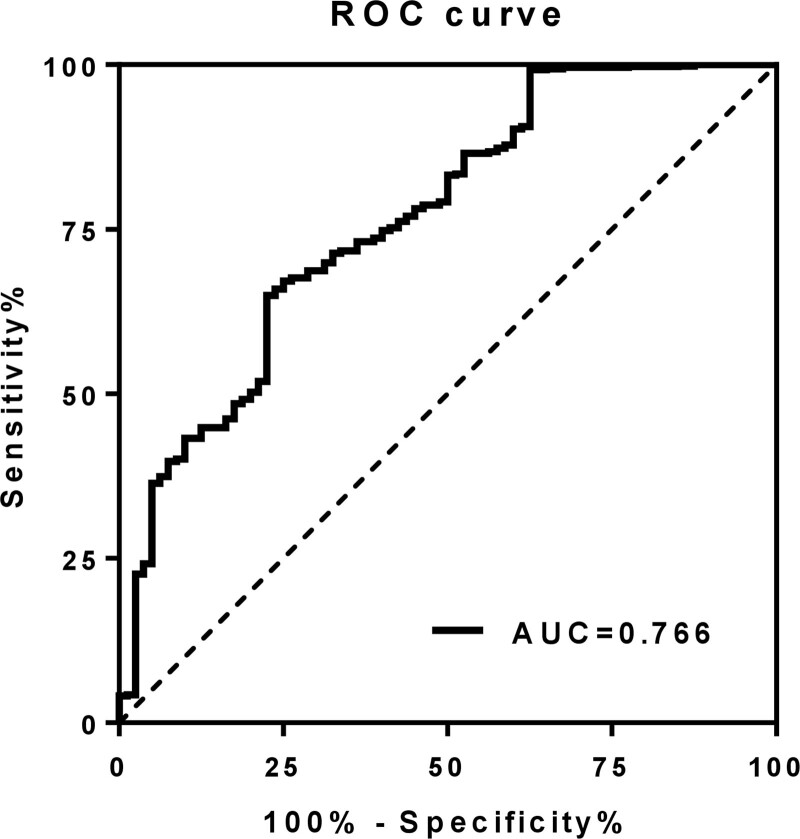
The receiver operating characteristic (ROC) curve to explore the diagnostic value of the aspartate and alanine aminotransferase (AST/ALT) ratio for major adverse cardio-cerebral events.

As shown in Figure 2, the ROC curve showed that the optimal cutoff point of the AST/ALT ratio to predict MACCE was 1.29, with an area under the curve of 0.77 (95% confidence interval 0.69–0.84, *P* < .001). Meanwhile, the sensitivity and specificity of MACCE prediction in the AST/ALT ratio of 1.29 were 77.5% and 65.1%, respectively.

## 4. Discussion

To our knowledge, this is the first study exploring the impact of the AST/ALT ratio on outcomes of UA patients undergoing PCI. The major results were as follows: (1) Patients with a high AST/ALT ratio were older, had more extensive CAD and myocardial injury, and poor nutritional status; (2) patients with a high AST/ALT ratio had an increased risk of MACCE and TVR during 13 months of follow-up; (3) notably, the AST/ALT ratio was associated with MACCE in a multivariate analysis adjusting for potential covariates (e.g., Gensini score); importantly, the prognosis predicting value was enhanced using the AST/ALT ratio (compared with AST or ALT) beyond traditional risk factors; (4) the optimal threshold of the AST/ALT ratio to predict MACCE was 1.29, with a sensitivity and specificity of 77.5% and 65.1%, respectively.

Since the liver receives almost 25% of the total cardiac output, it is sensitive to hemodynamic alterations.^[17]^ Therefore, liver dysfunction (i.e., elevated serum transferase levels) is commonly seen in patients with CVD.^[18]^ It is reasonable to speculate that the AST/ALT ratio might be a prognostic marker of CVD patients. Recently, in a large cohort of primary care patients in the UK (n = 29,316), the AST/ALT ratio was significantly associated with the increased risk of developing CVD in men over 10 years follow up.^[19]^ Additionally, the predictive value of AST/ALT ratio for cardiovascular mortality has been found in a longitudinal cohort with 3494 Japanese community residents during 10-year follow-up.^[20]^ Similarly, the prognostic value of high AST/ALT ratio has also been confirmed in Chinese patients with hypertension and peritoneal dialysis.^[21–23]^

To further expand these findings, we performed this study in UA patients undergoing PCI. We found that patients with the high AST/ALT ratio were more likely to have more frailty, severe CAD, and myocardial injury or necrosis. These results were consistent with previous findings that elevated AST/ALT ratio was associated with higher brain natriuretic peptide levels in a large longitudinal cohort study with community-based population.^[20]^ Moreover, Steininger et al^[24]^ showed that the AST/ALT ratio was positively correlated with age, the number of diseased vessels, and the highest cardiac troponin T levels in ST-segment elevation myocardial infarction patients.

Notably, in the present study, we found that the high AST/ALT ratio was significantly (in particularly >1.29) associated with the cumulative incidence of MACCE at 13 months, which was primarily driven by TVR. Importantly, the prognostic value of AST/ALT ratio was independent of age, hemoglobin, extent of CAD, and prior myocardial revascularization. These findings have significantly clinical importance because the UA patients who with increased risk of MACCE could be identified on admission using the easily accessible aminotransferase ratio. Meanwhile, physicians should focus on these high-risk populations with regard to optimizing PCI procedure, medications at discharge, and follow-up plan in order to reduce the incidence of TVR.

Potential explanations for the link between the high AST/ALT ratio and the incidence of TVR are as follows. Atherosclerosis is the major mechanism of myocardial ischemia that requires PCI. The high AST/ALT ratio is associated with the numerous risk factors for atherosclerosis (e.g., metabolic syndromes and chronic kidney disease^[19,23,25]^) and incidence of arteriosclerosis.^[22,26,27]^ Furthermore, the AST/ALT ratio is positively correlated with serum levels of interleukin-4, interleukin-6, tumor necrosis factor-α, and reactive oxygen species.^[28,29]^ Systemic inflammation and oxidative stress are considered as the major pathophysiology of the in-stent restenosis and stent thrombosis in the coronary arteries.^[30–33]^ However, these hypotheses should be further investigated and confirmed in dedicated experiments using appropriate in vitro and in vivo models.

This study has limitations. (1) Due to designs of the inherent observational study, potential bias and heterogeneities existed, and thus no causal relationship could be determined. (2) This study cohort was derived from a single center in China, so it may be inappropriate to apply the main findings of the present study to other ethnic groups or populations with different medical systems. (3) Aminotransferase was measured only at baseline, the relationship between the changes of the AST/ALT ratio during hospitalization or follow-up and MACCE was uncertain.

## 5. Conclusions

In UA patients undergoing PCI, a high AST/ALT ratio (i.e., >1.29) on admission is associated with an increased risk of MACCE, which is primarily driven by repeat revascularization. More prospective multicenter studies are needed to confirm the prognostic value of this aminotransferase index.

## Author contributions

**Conceptualization:** Li Zhang.

**Formal analysis:** Dong Lv.

**Investigation:** Dong Lv, Yanfu Guo.

**Visualization:** Xia Li.

**Writing – original draft:** Yanfu Guo, Xia Li.

**Writing – review & editing:** Li Zhang.
